# Supplementary material for: The Evolution and Characterization of the RNA Interference Pathways in Lophotrochozoa
Source: Genome Biol Evol. 2024 May 7;16(5):evae098. doi: 10.1093/gbe/evae098 (PMC11114477; doi:10.1093/gbe/evae098)
Supplement: evae098_Supplementary_Data [file evae098_supplementary_data.zip › Supplementary_materials.zip!/selected_miRNAs/NoGe_selected_miRNAs/mirdeep_pdf_files/NMRB01005527.1_Notospermus_geniculatus_strain_Ushimado_scaffold5527,_whole_genome_shotgun_sequence_47684.pdf]

Provisional ID	:	NMRB01005527.1_Notospermus_geniculatus_strain_Ushimado_scaffold5527,_whole_genome_shotgun_sequence_47684
Score total	:	34.8
Score for star read(s)	:	3.9
Score for read counts	:	29.2
Score for mfe	:	2.3
Score for randfold	:	0
Score for cons. seed	:	-0.6
Total read count	:	69
Mature read count	:	52
Loop read count	:	0
Star read count	:	17

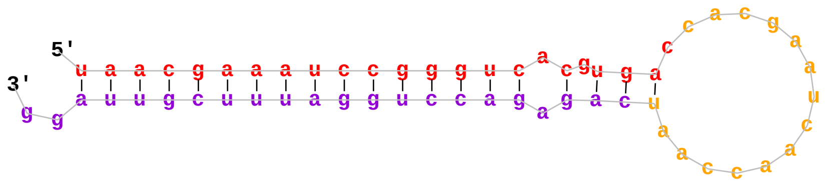

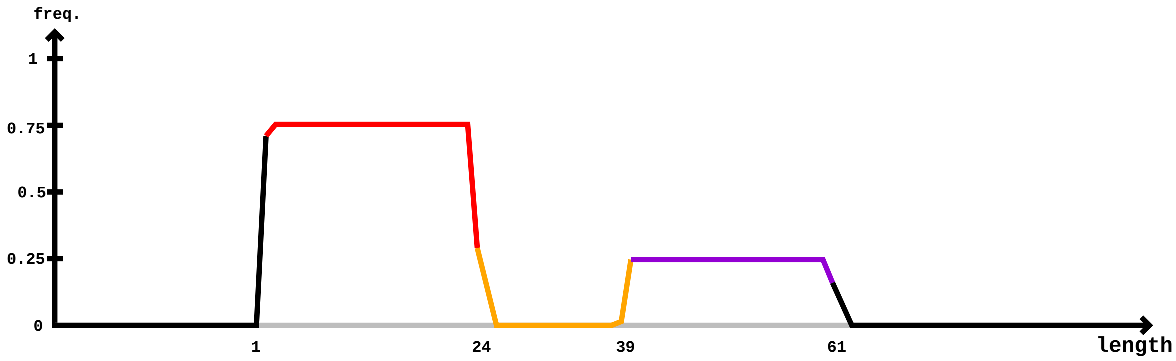


```
                    Mature                               Star
5'- cgacguaccuguaccuggccuaacgaaauccgggucacgugaccacgaaucaaccaaucagagaccuggauuucguuaggacagguacaggucaucgccaucuuggaaccgg   -3'    obs
    cgacguaccuguaccuggccuaacgaaauccgggucacgugaccacgaaucaaccaaucagagaccuggauuucguuaggacagguacaggucaucgccaucuuggaaccgg          exp
    (((.(.(((((((((((.((((((((((((((((((.(.(((...............)))).)))))))))))))))))).)))))))))))).)))(((...)))......    reads    mm    sample
    ....................uaaGgaaauccgggucacguga......................................................................    2        1     Fm3
    ....................uaacgaaauccgggucacgugac.....................................................................    12       0     Fm3
    ....................uaacgaaauccgggucaGgugac.....................................................................    1        1     Fm3
    .....................aacgaaauccgggucacgugac.....................................................................    1        0     Fm3
    ..........................................................cagagaccuggauuucguuag.................................    1        0     Fm3
    ..........................................................cagagaccuggauuucguuaggU...............................    1        1     Fm3

    ....................uaacgaaauccgggucacgugG......................................................................    8        1     Mm2
    ....................uaacgaaauccgggucacguga......................................................................    12       0     Mm2
    ....................uaacgaaauccgggucGcguga......................................................................    1        1     Mm2
    ....................uaacgaaauccgggucacgugac.....................................................................    5        0     Mm2
    .....................aacgaaauccgggucacgugG......................................................................    1        1     Mm2
    .....................aacgaaauccgggucacgugac.....................................................................    1        0     Mm2
    .........................................................ucagagaccuggauuucguuag.................................    1        0     Mm2
    ..........................................................cagagaccuggauuucguuaU.................................    2        1     Mm2
    ..........................................................cagagaccuggauuucguuag.................................    2        0     Mm2
    ..........................................................cagagaccuggauuucguuagU................................    1        1     Mm2
    ..........................................................cagagaccuggauuucguuagA................................    2        1     Mm2
    ..........................................................cagagaccuggauuucguuagg................................    5        0     Mm2
    ..........................................................cagagaccuggauuucguuaggU...............................    1        1     Mm2
    ..........................................................cagagaccuggauuucguuagga...............................    1        0     Mm2

    ....................uaacgaaauccgggucacguga......................................................................    7        0     Mm1
    ....................uaacgaaauccgggucacgugG......................................................................    1        1     Mm1
```
